# Increased MDSC Accumulation and Th2 Biased Response to Influenza A Virus Infection in the Absence of TLR7 in Mice

**DOI:** 10.1371/journal.pone.0025242

**Published:** 2011-09-23

**Authors:** Victoria Jeisy-Scott, William G. Davis, Jenish R. Patel, John Bradford Bowzard, Wun-Ju Shieh, Sherif R. Zaki, Jacqueline M. Katz, Suryaprakash Sambhara

**Affiliations:** 1 Influenza Division, National Center for Immunization and Respiratory, Centers for Disease Control and Prevention, Atlanta, Georgia, United States of America; 2 Division of High-Consequence Pathogens and Pathology, National Center for Emerging and Zoonotic Infectious Diseases, Centers for Disease Control and Prevention, Atlanta, Georgia, United States of America; 3 Emory University, Atlanta, Georgia, United States of America; University of Georgia, United States of America

## Abstract

Toll-like receptors (TLRs) play an important role in the induction of innate and adaptive immune response against influenza A virus (IAV) infection; however, the role of Toll-like receptor 7 (TLR7) during the innate immune response to IAV infection and the cell types affected by the absence of TLR7 are not clearly understood. In this study, we show that myeloid derived suppressor cells (MDSC) accumulate in the lungs of TLR7 deficient mice more so than in wild-type C57Bl/6 mice, and display increased cytokine expression. Furthermore, there is an increase in production of Th2 cytokines by TLR7^-/-^ compared with wildtype CD4+ T-cells *in vivo*, leading to a Th2 polarized humoral response. Our findings indicate that TLR7 modulates the accumulation of MDSCs during an IAV infection in mice, and that lack of TLR7 signaling leads to a Th2-biased response.

## Introduction

The innate immune system is the frontline for host defense against pathogens and is evolutionarily conserved among many organisms [1], [2], [3]. The innate immune system recognizes a diversity of conserved motifs, referred to as pathogen associated molecular patterns (PAMPs), through a number of immune sensors, known as pattern recognition receptors (PRRs). Mammalian PRRs are found in nearly every cell of the body and are distributed throughout the cell, thus casting a wide net to detect invading pathogens [4], [5], [6], [7]. Some well studied families of PRRs include collectins, pentraxins, Toll-like receptors, C-type lectin receptors, and retinoic acid inducible gene I (RIG-I)-like receptors [3], [4], [5], [6], [7], [8]. These PRRs recognize PAMPs such as bacterial cell wall components, flagellar proteins, and nucleic acids. Viral replication produces distinct nucleic acids not commonly found in mammalian cells, such as double stranded, uridine-rich or unmodified RNA, as well as DNA found outside of the nucleus. These unusual nucleic acids are recognized by TLRs 3, 7, and 9, or RIG-I [7], [9], [10].

Influenza A virus (IAV) remains a major public health burden. Hence, it is important to understand how the innate immune response programs the resulting protective adaptive immune response to IAV [11], [12]. IAV has a single-stranded, negative-sense, segmented RNA genome. TLR3, which recognizes double stranded RNA (dsRNA), has been shown to either play a minor role or contribute negatively to the inflammatory response to IAV infection [13], [14]. TLR7 senses single-stranded RNA (ssRNA) within an endosome, whereas RIG-I detects ssRNA in the cytoplasm; both have been shown to be instrumental in the induction of a protective immune response to IAV infection [15], [16], [17], [18], [19], [20], [21]. Myeloid differentiation primary response gene 88 (MyD88) and interferon-β promoter stimulator 1 (IPS-1), the adapter proteins downstream of TLR7 and RIG-I, are redundant in their ability to activate type I interferons (IFNα/β) in response to acute IAV infection both *in vitro* and *in vivo* [15]. These adaptors, however, play different roles in the resulting adaptive immune response. While mice lacking MyD88 had decreased levels of the Th1 polarized antibody IgG2a as well as a decreased CD4+ T-cell IFNγ response, IPS1^-/-^ mice and wild type mice had normal levels of IgG2a and IFNγ production [15]. Similar to MyD88^-/-^ mice, TLR7^-/-^ mice are deficient in IgG isotype switching of the humoral response [13], [18], [22], [23], [24]. Furthermore, MyD88 signaling is important for T-cell polarization of lymphocytes *in vitro*, as its absence leads to a Th2 bias four weeks post-infection (p.i.) [18]. Although changes in the adaptive response are evident, it is not clear whether the altered immune responses seen in the MyD88^-/-^ model are due to the inhibition of pathogen recognition by a TLR or to the inhibition of IL-1 signaling. The specific contribution of TLR7 to the regulation of the innate immune response to IAV infection is still unclear.

To answer this question, we investigated the innate immune response in B6 and TLR7^-/-^ mice during acute IAV infection and observed subsequent differences in the adaptive immune response. We found that lack of TLR7 leads to the accumulation of Gr1^+^CD11b^+^F4/80^+^ monocytes, otherwise known as myeloid derived suppressor cells (MDSC) in the lungs [25], [26]. The accumulation of MDSCs in TLR7^-/-^ mice during IAV infection was associated with the Th2 polarization of CD4+ T-cells and IgG isotypes.

## Results

### TLR7^-/-^ mice show increased morbidity, but similar lung viral titers following IAV infection compared to wild type mice

**Figure 1:**
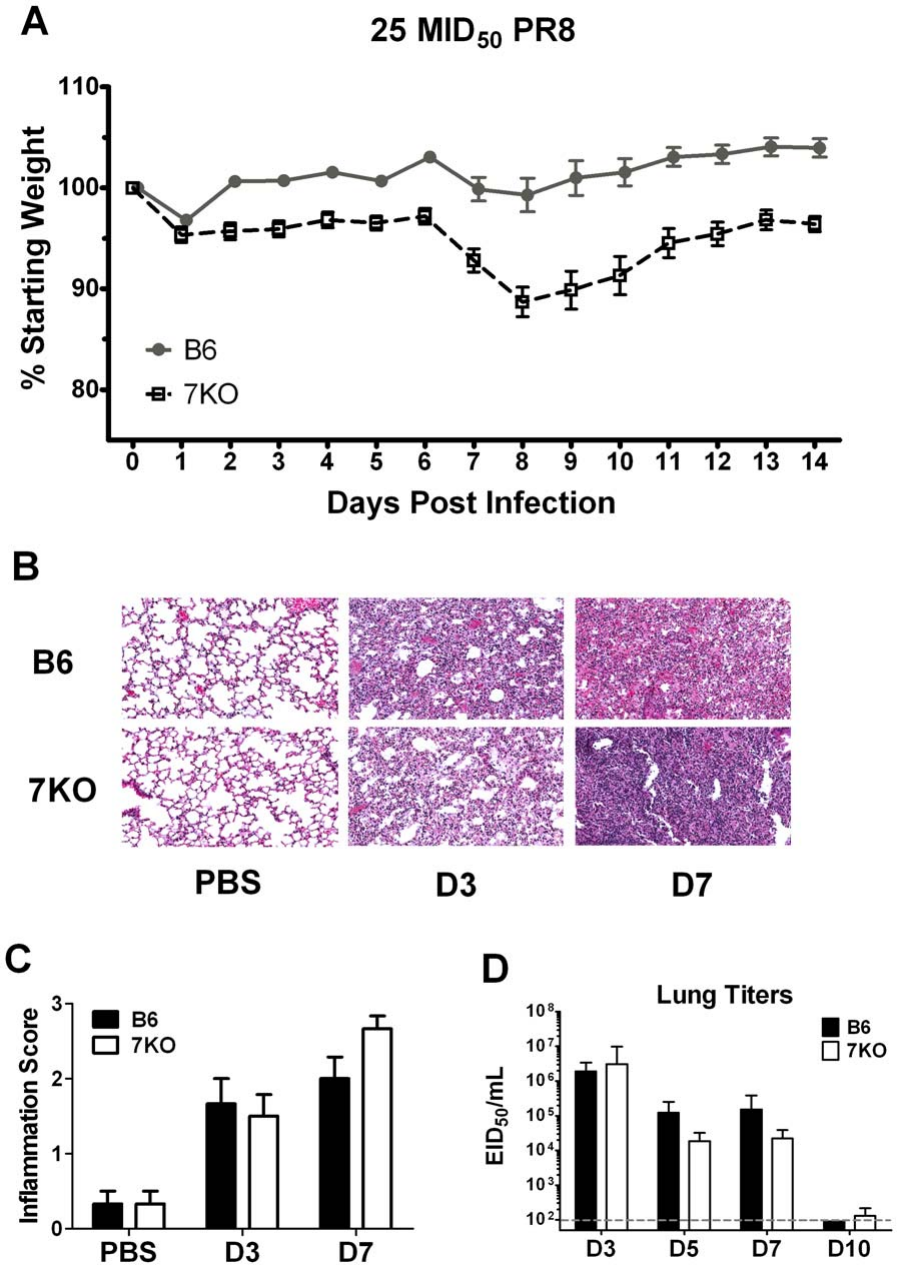
TLR7^-/-^ mice exhibit increased influenza-induced morbidity. B6 and TLR7^-/-^ mice were infected with 25 mID_50_ of A/PR8/34 (PR8) virus and monitored daily. (A) Mice (n≥13 mice for each group) were weighed individually. Displayed is the percent of starting weight averaged for each group. Significant differences were seen between groups on days 2 & 3 (p<0.01), day 4 (p<0.05), and days 6–14 (p<0.001) as determined by a Two-way ANOVA. (B) Three mice per group per day per treatment were sacrificed and lung samples collected for histology days 3 and 7 p.i. Displayed is a representative H&E stained section of three individual mice with original magnification 10X. (C) An inflammation score was determined by a pathologist of each individual mouse. These scores were combined and displayed on a scale from 0–3; 0 being no inflammation, 3 being the most intense inflammation. (D) At indicated days p.i. mice (n≥5) were sacrificed, lungs were homogenized, and viral titers determined as described in the materials and methods.

To examine the role of TLR7 in the innate immune response to IAV infection, B6 and TLR7^-/-^ mice were infected with a sub-lethal dose of PR8 and monitored for morbidity and mortality for two weeks. As expected, both strains of mice lost body weight on day one p.i. due to anesthesia. While B6 mice rapidly regained their pre-infection body weights, TLR7^-/-^ mice maintained only 95% of their original body weight for the first week. On day 7 p.i., TLR7^-/-^ mice began showing signs of morbidity, including lethargy and ruffled fur. They also experienced more substantial weight loss which peaked with a mean maximum weight loss of 11.3% on day 8 p.i. (Figure 1a). These mice did not show signs of recovery until 10 days p.i. B6 mice did not show signs of morbidity at any time during the course of this study (Figure 1a).

Histopathological evaluation of the lung tissues shows varying degrees of inflammation in different groups of experimental animals (Figure 1b). No prominent inflammation was observed in the lungs of the mock infected (PBS) control mice. The inflammatory cells observed in the lungs of infected animals were mainly composed of lymphocytes, plasma cells, and macrophages in peribronchiolar areas and in alveoli. No significant differences in inflammation were seen in mice harvested on day 3 p.i. between the two groups. The degree of inflammation in the lung tissues was more intense on day 7 than on day 3 in both B6 and TLR7^-/-^ mice. Furthermore, a more intense inflammation was observed in TLR7^-/-^ mice compared to B6 mice on day 7 (Figure 1 b, c).

Because it is possible that the increased morbidity of TLR7^-/-^ mice could be due to differences in viral burden and/or kinetics of viral clearance, viral titers were quantified in the lungs on days 3, 5, 7, and 10 p.i. (Figure 1d). Although viral titers were modestly lower in TLR7-/- mice on days 5 and 7 p.i., these differences were not statistically significant. These results indicate that the increased morbidity observed in TLR7^-/-^ mice is not due to increased viral replication in the lungs of the infected mice.

### IAV infection in TLR7^-/-^ mice does not result in hypercytokinemia

**Figure 2:**
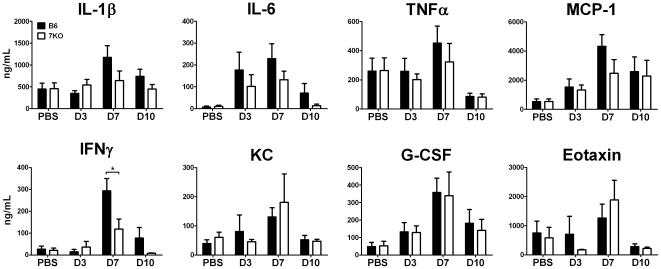
The absence of TLR7 does not significantly alter influenza-induced inflammatory cytokine profiles. On the indicated days p.i., cytokine levels within homogenized lung samples were determined using a bead-based multiplex immunoassay as described in the materials and methods. The data represent a combination of n≥5 individual mice from two independent experiments.

A potential cause of immunopathology during IAV infection is the excess production of inflammatory mediators, known as hypercytokinemia or a “cytokine storm”, as observed in cases of highly pathogenic avian influenza (H5N1) viral infections in mice [27], [28]. To investigate this possibility that increased levels of inflammatory cytokines were present in the lungs of TLR7^-/-^ mice, we harvested lungs from PR8 infected B6 and TLR7^-/-^ mice on days 3, 7, and 10 p.i. and measured the levels of cytokines produced. Levels of inflammatory cytokines and chemokines in TLR7^-/-^ and B6 mice were generally similar on day 3 p.i. However, on day 7 p.i., a timepoint of increase morbidity in TLR-/- mice, these mice produced lower levels of IL-1β, IL-6, TNFα, and MCP-1 (Figure 2). We also observed a 60% decrease in the levels of the Th1 cytokine IFNγ in TLR7^-/-^ mice compared to B6 at this time point (Figure 2). KC, G-CSF, and Eotaxin levels were similar between the control and TLR7^-/-^ mice. These results suggest that TLR7^-/-^ morbidity following IAV infection is not due to hypercytokinemia.

### TLR7^-/-^ mice accumulate increased numbers of Gr1^+^CD11b^+^ monocytes, neutrophils and dendritic cells to the lungs during IAV infection

**Figure 3:**
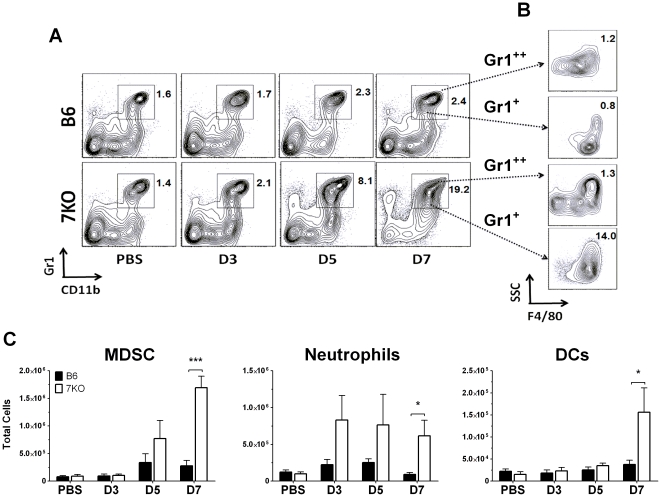
Increased recruitment of Gr1^+^ CD11b^+^ cells to the lungs of TLR7^-/-^ mice. On indicated days p.i., lungs from PR8 infected mice or mock infected PBS control mice (n≥5) were processed; infiltrating cells were isolated and stained for analysis by flow cytometry. (A) CD3^-^CD19^-^ cells were measured for their dual expression of Gr1 and CD11b. (B) These cells were further gated by F4/80 expression to separate neutrophils (SSC^+^F4/80^-^) from MDSCs (SSC^low^F4/80^+^). (A, B) Numbers indicated on gates of dot plots represent % total cells. (C) Total cell numbers from two independent experiments were calculated for MDSCs (Gr1^+^CD11b^+^F4/80^+^SSC^low^), neutrophils (Gr1^+^CD11b^+^F4/80^+^SSC^high^), and dendritic cells (CD11c^+^Gr1^-^F4/80^-^).

We next investigated whether the absence of TLR7 affected the types of cells recruited to the lungs during the first week of IAV infection. No differences were seen in the recruitment of lymphocytes between TLR7^-/-^ and B6 mice on any of the days tested (Figure S2a). However, on day 7 p.i. the lungs of TLR7^-/-^ mice had an 8-fold increase in the number of Gr1^+^, CD11b^+^ cells (Figure 3a). 73% of these cells were F4/80^+^, SSC_low,_ Gr1_mid_ (Figure 3b-c). Cells with these phenotypic markers are known to be MDSCs [25], [26], [29] or inflammatory monocytes (iMo) [30], [31]. Neutrophils and dendritic cells (DC) were also detected at significantly higher numbers in TLR7^-/-^ mice (Figure 3c). When day 10 was examined, many of the innate cell infiltrates decreased from day 7, while T-cells and B-cells increased in prevalence (Figure S2b). No statistically significant changes were seen between the cell types, except for a relatively lower accumulation of B-cells in the TLR7^-/-^ mice. Our findings, consistent with those reported earlier [15], [22], [32], [33], indicate that TLR7 is not essential for the very early innate response to IAV infection. Our data suggest that TLR7 is involved in the second wave of the innate immune response, mainly through the recruitment and/or activity of the pulmonary leukocytes. Furthermore, TLR7 may play a specific role in the accumulation of MDSCs in response to IAV infection distinct from that of the other TLRs and MyD88 dependent pathways [34].

### Gr1^+^CD11b^+^ monocytes from TLR7^-/-^ mice are IL-10, TNFα dual producing cells that influence the cytokine production of T-cells *in vitro*

**Figure 4:**
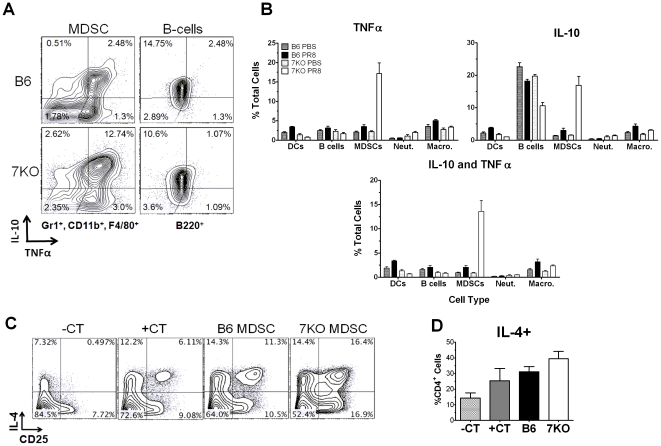
Functional analysis of lung-derived MDSCs shows greater activity in TLR7^-/-^ mice. (A,B) Lungs were harvested and processed from PR8 infected and PBS mock infected mice (n = 3 for each group) 7 days p.i. Cells were stained for surface antigens and intracellular cytokines to be analyzed by flow cytometry. Individual cell types were gated and subsequently analyzed for the expression of IL-10 and TNFα. (B) Percent of total cells (n = 3 lungs) expressing IL-10, TNFα, or both is shown. (C,D) MDSCs from PR8 infected B6 or TLR7^-/-^ mice were co-cultured with OT-II T-cells and peptide pulsed APCs. T-cells were stained for intercellular cytokine expression of IL-4. Also included were T-cells co-cultured with peptide pulsed APCs only (+CT) and T-cells co-cultured with APCs without peptide pulsing (-CT). (C) Dot pots of a representative experiment of three independent experiments are presented. (D) Data from the combination of the three independent *in vitro* experiments was combined displaying the %CD4+ T-cells expressing IL-4.

MDSCs are known to have both inhibitory and inflammatory characteristics depending on the model and the circumstance, which is why a phenotypically identical cell type has been referred to as either MDSC or iMo [35], [36]. We wanted to determine if the Gr1^+^CD11b^+^ cells that were accumulating in the lungs of TLR7^-/-^ mice during IAV infection were multifunctional, similar to those observed in other models [37]. More than 15% of the total cells in the lungs of TLR7^-/-^ mice were MDSCs producing IL-10 (Figure 4a, b) on day 7 p.i. Not only were these MDSCs producing IL-10, but the majority of them (80%) were also co-expressing TNFα (Figure 4a, b). MDSCs expressing both cytokines were also observed in control B6 mice (2.1% of total lung cells), however they were lower in numbers when compared to TLR7^-/-^ mice (Figure 4a, b).

Next, we determined the functionality of these MDSCs by assessing their influence on the activation of T-cells to a novel antigen. MDSCs were purified from either B6 or TLR7^-/-^ mice 7 days p.i. and co-cultured with transgenic OT-II T-cells, along with OT-II peptide pulsed APCs. After 24 hours in culture, ICCS was performed. Addition of MDSCs from both B6 and TLR7^-/-^ mice induced increased expression of IL-4 from CD3^+^CD4^+^ cells compared to peptide pulsed APCs alone (Figure 4c, d). However, IL-4 production was further increased in the wells containing TLR7^-/-^ MDSCs (Figure 4c, d). Approximately 16% of the IL-4 producing cells in the TLR7^-/-^ cultures were also activated, based on their up regulation of CD25 (Figure 4c). Taken together, these results suggest that TLR7 not only affects the accumulation of MDSCs at the site of infection, but can also modulate their ability to influence the subsequent T-cell response.

### Evidence of increased Th2 polarization of T-cells in both the MLNs and lungs of TLR7^-/-^ mice

**Figure 5:**
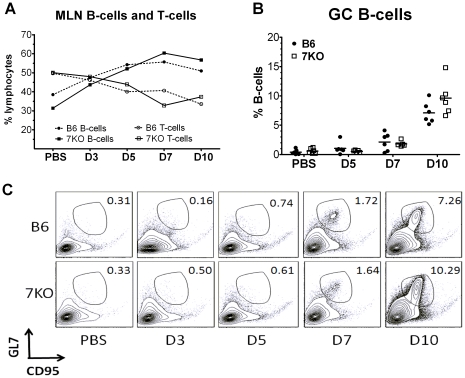
Increased expansion of germinal center B cells in TLR7^-/-^ mice. At indicated day p.i., MLN were harvested (n≥5 animals) and stained for surface antigens. (A) Changes in the relative number of B and T cells as a % of total lymphocytes are displayed over time. (B, C) B-cell germinal center activation was measured by % of B-cells co-expressing GL7 and CD95. (C) A representative dot plot and (B) a combination of all experiments are displayed.

Previously, it was shown that the MyD88 signaling pathway is important for the adaptive immune response to IAV [13], [15], [34], but the specific role that TLR7 plays in this response is still unclear. We next examined if there were differences in the activation of B-cells in the mediastinal lymph nodes (MLN), where the B-cells first encounter antigen. There was an increase in the relative number of B-cells in the MLN, increasing steadily from day 3 through 7 p.i. TLR7^-/-^ mice showed a greater expansion of B-cells at day 7 and 10 compared to B6 mice, although these differences were not statistically significant (Figure 5a). Concordant with the overall increase in B-cell numbers, was an increased expansion of GL7^+^ CD95^+^ germinal center B-cells in TLR7^-/-^ mice compared to B6 mice (Figure 5 b, c). One explanation for this observation would be the presence of increased numbers of T-helper cells expressing the B-cell growth factor IL-4, a consequence of Th2 polarization.

**Figure 6:**
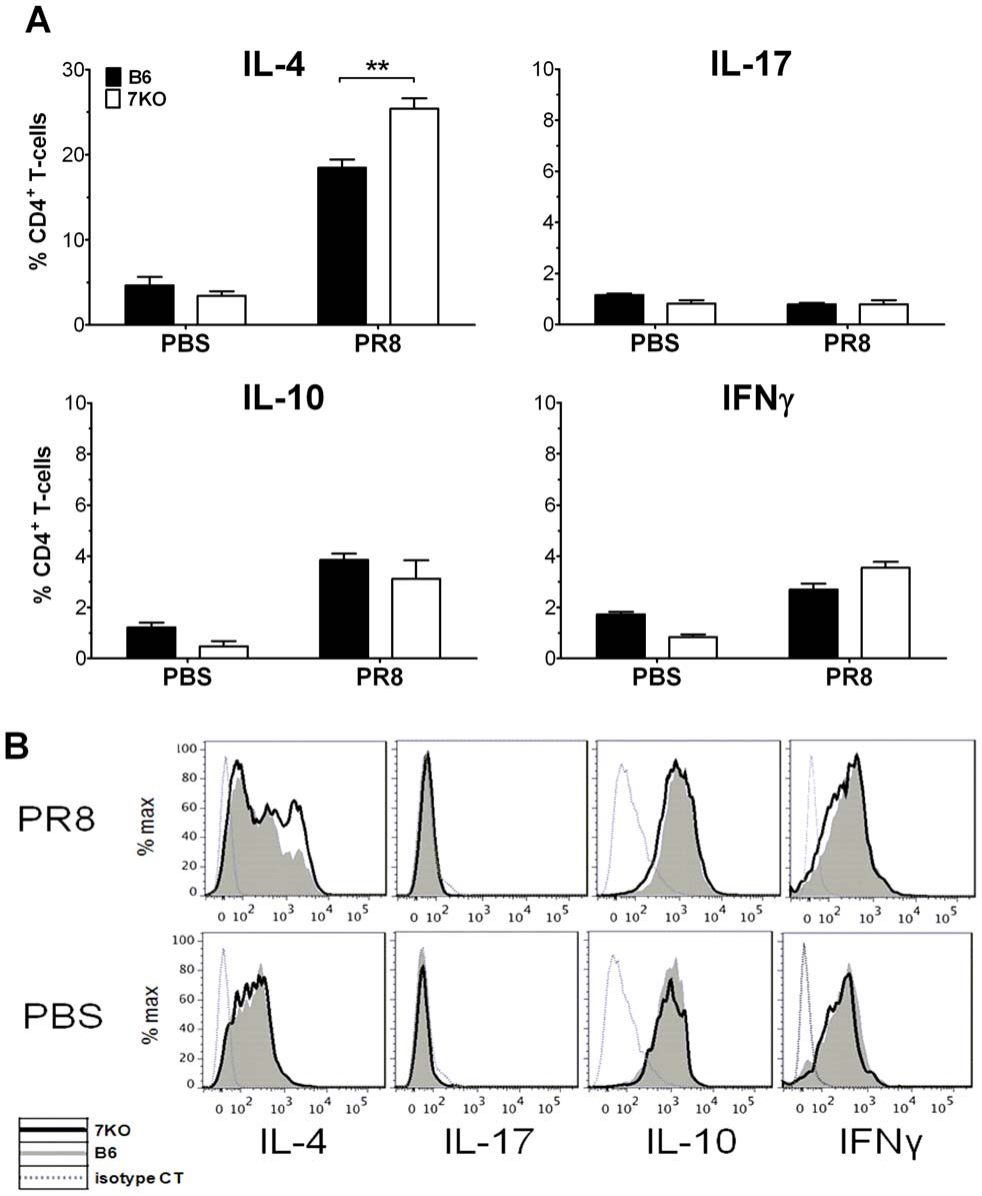
Absence of TLR7 leads to increased numbers of IL-4 producing CD4^+^ T cells. 10 days p.i., lungs (n≥6 animals) were harvested, infiltrating cells were stained with appropriate fluorochrome conjugated antibodies and ICCS performed as described in materials and methods. (A) Data from for each animal was combined as a percentage of CD4+ T-cells expressing either IL-4, IL-17, IL-10 or IFNγ. (B) One representative histogram from each group is also shown.

**Figure 7:**
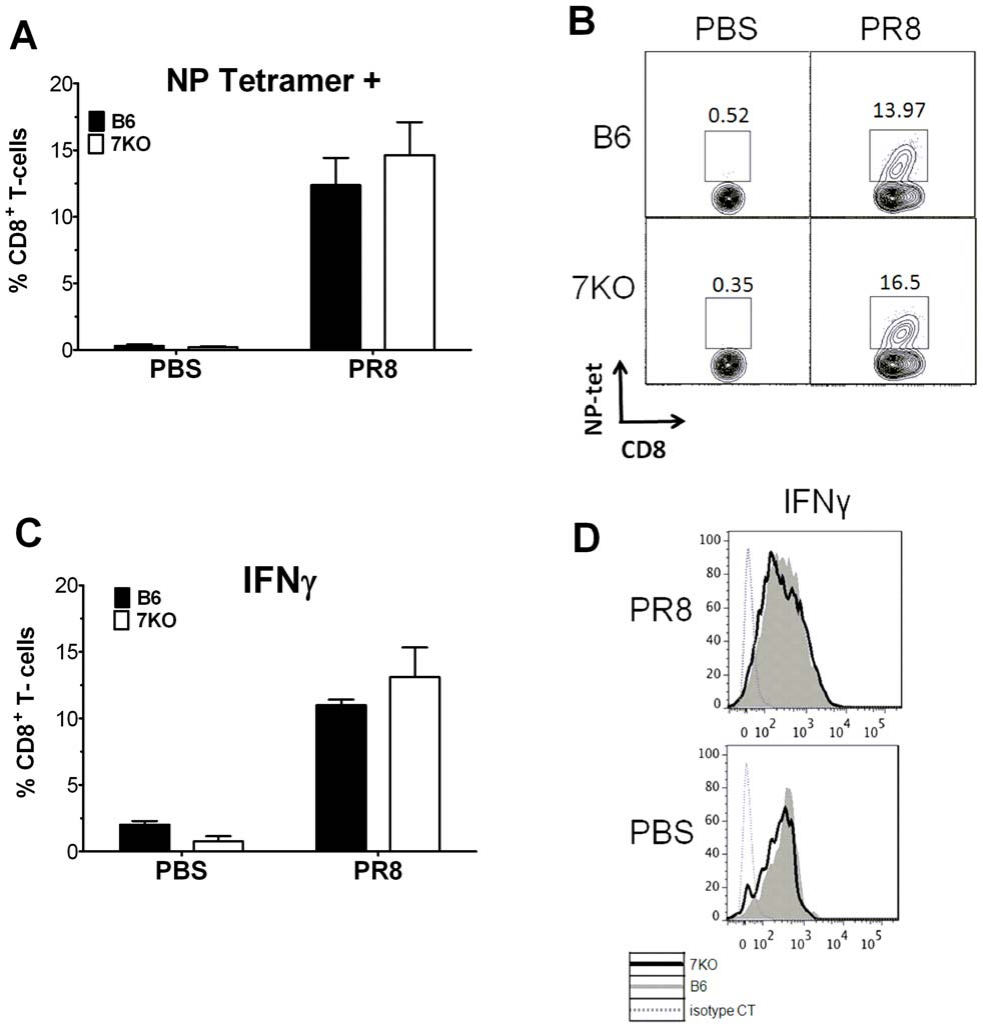
The frequency and IFN-γexpressing NP-specific CD8^+^ T cells are not affected in TLR7^-/-^ mice. 10 days p.i., lungs (n≥6 animals) were harvested, infiltrating cells were stained with appropriate fluorochrome conjugated antibodies and ICCS performed as described in materials and methods. (A) Data from for each animal was combined showing the % NP-tetramer positive CD8+ T-cells. (B) One representative histogram from each group is also shown. (C) Data from for each animal was combined as a percentage of CD8+ T-cells expressing IFNγ. (D) One representative histogram from each group is also shown.

To investigate further the possibility of increased Th2 polarization, we compared T-cells from the lungs of TLR7^-/-^ mice with those from B6 mice on day 10 p.i. We found a 40% increase in the numbers of IL-4 producing CD4+ T-cells in the lungs of TLR7^-/-^ mice than in B6 mice (Figure 6a, b). However, we did not see significant increases in the number of IL-17, IL-10, or IFNγ producing T-cells (Figure 6 a, b). There was also no change in the production of these cytokines in splenic T-cells (Figure S3a). When we examined CD8+ T-cells, we saw no differences in frequency or function of antigen specific CD8+ T-cells (Figure 7a, d) as reported previously by others [13], [15], [34]. Taken together, our findings suggest that TLR7^-/-^ mice have a Th2 T-cell bias in response to IAV.

### B-cells in TLR7^-/-^ mice produce more IgG1 than IgG2a during the early adaptive immune response to IAV

**Figure 8:**
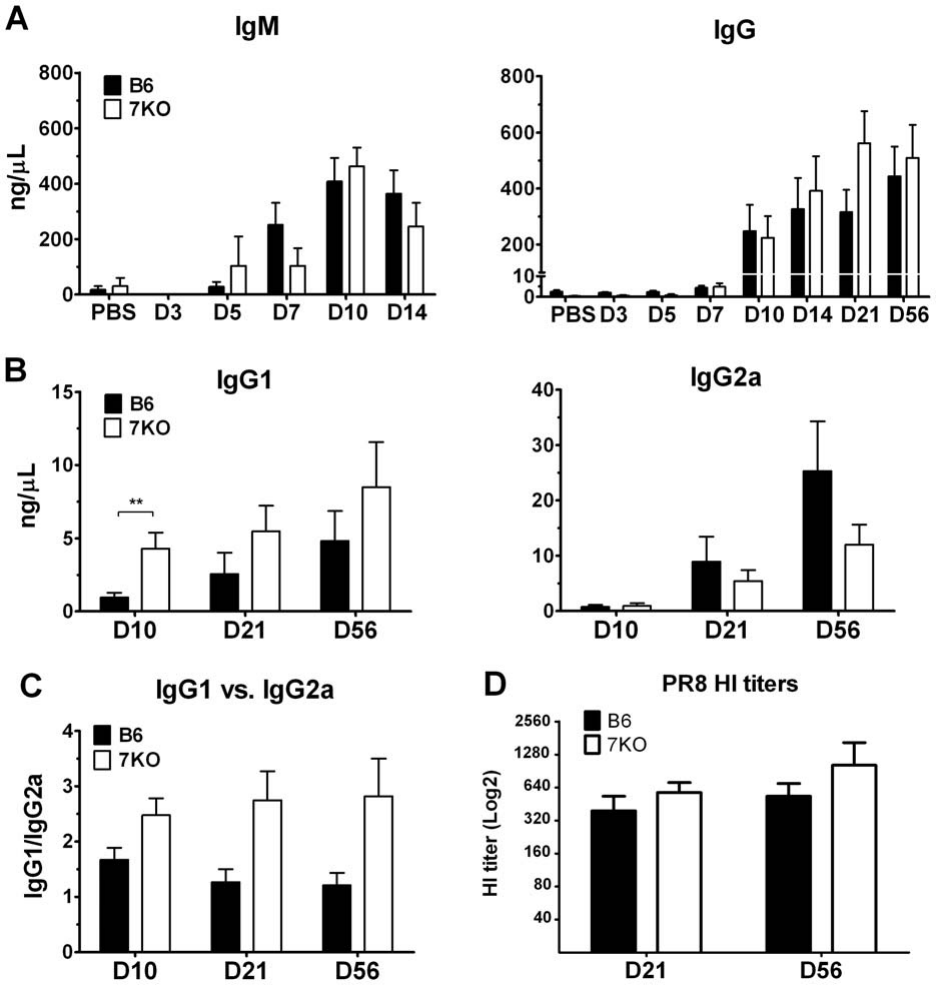
Th2 polarization in TLR7^-/-^ mice alters IgG isotype switching of influenza-specific antibodies. Sera were collected from B6 and TLR7^-/-^ infected mice (n≥6 per group) at indicated days p.i. PR8 specific (A) IgM or IgG antibodies were measured by ELISA. Sera from additional animals were collected over time (n = 10 per group) and PR8-specific (B) IgG1 and IgG2a were measured. Antibody concentration was determined based on a standard curve of their respective immunoglobulin type. (C) A ratio of IgG1 **versus (**vs.) IgG2a prevalence was determined by dividing a sample’s IgG1 A450 absorbance by its IgG2a absorbance. (E) HI titers were determined as described in the materials and methods.

To further investigate the possible Th2 bias of the adaptive response of TLR7^-/-^ mice, we examined the IgG subclass distribution of PR8 specific serum antibodies. A Th2 polarized response produces increased levels of IgG1, where a Th1 polarized response induces increased levels of IgG2a [13], [38], [39]. Such a Th2 bias has been previously reported in MyD88^-/-^ mice [13], [15]. Although both B6 and TLR7^-/-^ mice had similar levels of HI antibody titers, as well as PR8 specific IgM and IgG (Figure 8a,b, d), the IgG isotypes differed on various days p.i. (Figure 8b). We found that TLR7^-/-^ mice produced relatively higher levels of IgG1, where B6 mice produced relatively higher levels of IgG2a (Figure 8 b, c). These results further demonstrate that the lack of TLR7 signaling leads to a Th2 polarized environment in response to IAV infection.

## Discussion

The main function of the innate immune system is not only to limit the early replication and spread of the invading pathogen, but also to initiate an adaptive response to clear the infection and establish long-lasting immunological memory. TLR7 is one of the major RNA sensing PRRs involved in the detection of IAV infection. Currently, it is understood that TLR7’s downstream signaling pathway leads to the activation of proinflammatory cytokines and IFNs; other RNA sensing PRRs, such as RIG-I, activate similar responses. Previously, it was shown that MyD88 signaling, not RIG-I signaling, plays a unique role in the CD4^+^ polarized response to IAV infection, but the mechanism of this was unknown. We demonstrate that the lack of TLR7 signaling significantly increases the accumulation of MDSCs at the site of infection. Because MDSCs have been previously shown to alter CD4+ T-cell polarization, we propose that MDSC recruitment is the mechanism by which TLR7 effects the resulting T-cell TH1/TH2 balance.

In a study by *Seo et al.,* MyD88 was shown to be required for protection from IAV infection, as MyD88^-/-^ mice displayed increased morbidity and increased viral titers when infected with PR8 [34]. MyD88^-/-^ mice were inhibited in their ability to recruit CD11b^+^ granulocytes, produce inflammatory cytokines, and Th1 cytokine production by CD4+ T cells following IAV infection when compared to B6 mice. A study by *Koyama et al.*, conversely, showed no changes in viral titer when MyD88^-/-^ mice were infected with A/New Caledonia/20/99. They demonstrated that RIG-I and MyD88 were redundant in their ability to induce the early IFNα/β response *in vivo* and *in vitro* [15]. However, they found changes in the IgG istotypes in MyD88^-/-^ mice following IAV infection with increased IgG1 with a concomitant decrease in IgG2a on day D10 [15]. These studies demonstrated that MyD88 signaling is instrumental in the shaping of not only the innate but also the adaptive responses to IAV.

MyD88 is the downstream adaptor not only for many of the TLRs, but is also downstream of IL-1 receptor signaling [40]. For this reason, it is difficult to ascertain if the phenotypes observed in MyD88^-/-^ mice are a result of dysfunctional IL-1 cytokine signaling or if they are due to inhibited viral recognition by a PRR. Like MyD88^-/-^ mice, IL-1^-/-^ mice display an increase in morbidity and lung viral titers in response to IAV [41]. Because of these similarities, it is possible that many of the effects observed in the MyD88^-/-^ model could be attributed to the inhibition of IL-1 signaling more so than the inhibition of TLR signaling. To better understand the effects of PRR specific recognition of IAV, independent of cytokine signaling inhibition, we utilized TLR7^-/-^ mice. We found that TLR7^-/-^ mice had increased morbidity, starting on day 7 p.i. (Figure 1a). In many viral infections, increased morbidity is caused by increased viral titers and/or cytokine storm [27], [28]. Previously, it was shown that absence of the inflammatory cytokines TNFα and IL-1 reduces the severity of H5N1 infection [42]. In our study, viral titers did not increase in the absence of TLR7 (Figure 1d). Also, many of the inflammatory cytokines measured were slightly decreased rather than showing signs of a cytokine storm (Figure 2). The only significant change in cytokines observed in the lungs was the decrease in IFNγ.

The relative decrease in inflammatory cytokines in the lungs did not have a detrimental effect the recruitment of myeloid cells (Figure 3a–e). This is contrary to what was observed in the MyD88^-/-^ and IL-1R^-/-^ models of IAV [34], [41]. Macrophage and neutrophil accumulation during IAV has been shown to play both a protective and pathogenic role depending on the magnitude of the cellular infiltrate. Depletion of neutrophils after a sub-lethal dose of IAV induces uncontrolled virus growth and lethality [43], [44]. In addition to hypercytokinemia, much of the pathology associated with highly pathogenic IAV infection is attributed to the recruitment of these myeloid cell types [27]. *Lin et al.* found that CCR2 dependent Gr1^+^CD11b^+^ iMo were the largest population recruited to the lungs during PR8 infection, and were responsible for much of the pathology associated with IAV in mice, but not in the control of virus replication [30]. In our study, we also show that the relatively large recruitment of Gr1^+^CD11b^+^ cells in TLR7^-/-^ mice was likely the major cause of morbidity observed with little or no effect on lung viral titers.

Gr1^+^CD11b^+^ cells are recruited to a site of inflammation in several models of inflammation [30], [35], [37], [45]. MDSCs have been described to be a Gr1^+^CD11b^+^ immature myeloid cell population derived from monocytes migrating out of the blood to the site of infection [29], [46]. It has been previously shown that the presence of MDSCs skews the immune response towards a Th2 response [37], [45], [47], [48]. This is mainly attributed to their ability to induce IL-10 and reactive oxygen species [47], [48] Like other groups, the TLR7^-/-^ MDSCs not only produced IL-10, but also TNFα (Figure 4a,b) [37]. Interestingly, TNFα has been shown to amplify the ongoing Th1 or Th2 response rather than favoring one over the other [49], [50]. The ability of TLR7^-/-^ MDSCs to coproduce IL-10 and TNFα may be one mechanism that encourages the Th2 bias observed in TLR7^-/-^ mice during IAV infection. On day 10 p.i., the lungs of TLR7^-/-^ mice had increased numbers of IL-4 producing CD4+ T-cells present (Figure 6). One month following infection with IAV, we found increased levels of IgG1 and decreased amounts of IgG2a in sera of TLR7^-/-^ mice (Figure 8), suggesting that TLR7^-/-^ mice do in fact display a Th2 bias during IAV infection. We hypothesize that this is due to the increased recruitment of MDSCs to the site of infection.

Interestingly, it has been shown in different models that MyD88 is required for the expansion of MDSCs *in vivo* [34], [37]. TLR7^-/-^ MDSCs, when compared to B6, not only show increased recruitment to the site of infection and secrete increased amounts of Th1 inhibiting IL-10 *in vivo*, but also increase the amount of IL-4 production from OT-II T-cells (Figure 3, 4). It is possible that while MyD88 is required for MDSC expansion, through either IL-1 signaling or another TLR becoming activated, TLR7 stimulation during IAV infection may be involved in the suppression of MDSC activity. TLR7 signaling could accomplish this through an inhibitory feedback mechanism or by inducing MDSCs to differentiate into another monocytic cell type. It has been previously shown that activation of MDSCs through either TLR9 or TLR4 can further differentiate MDSCs into a myeloid cell that no longer has tumor suppressor activity or Th2 allergy inducing polarization [26], [46]. If this is true in our model, the absence of TLR7 signaling in MDSCs may be responsible for further aggravating the Th2 bias observed due to increased MDSCs cytokine secretion and recruitment.

In the sepsis model, Gr1^+^CD11b^+^ MDSCs have been implicated for Th2 polarization observed [37]. The accumulation of MDSCs was dependent on MyD88 expression, as Gr1^+^CD11b^+^ cell recruitment to the spleen was inhibited during sepsis in MyD88^-/-^ mice [37]. CD11b^+^ cells in the spleens of wild type mice constitutively express TLR7, and TLR7’s expression is highly up regulated during sepsis. When mice were pre-treated with R-848 (a TLR7 ligand) before sepsis induction, the treated mice showed an increase in their ability to control bacterial load at the site of infection. This increase in pathogen control and inflammation demonstrate that TLR7 signaling will overcome the inhibitory phenotype predominant during sepsis [51].

**Figure 9:**
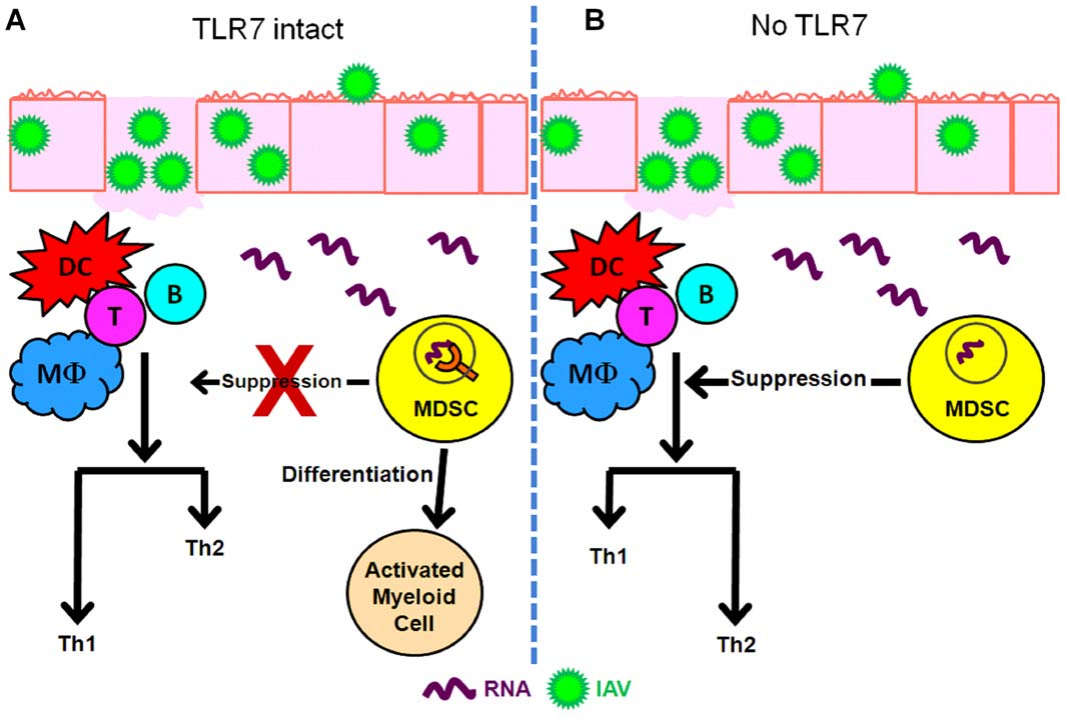
TLR7 inhibition of MDSC mediated Th1 suppression. (A) TLR7 functions to sense the presence of RNA in the endosomes following IAV induced inflammation. TLR7 stimulation may then activate the infiltrating MDSCs to further differentiate into other activated myeloid cell types, preventing the suppression of the ongoing Th1 polarized response. (B) When TLR7 is absent, MDSCs would not recognize the presence of RNA in the context of inflammation, leading to the recruitment of additional MDSCs and suppression of the Th1 mediated response, resulting in a Th2 bias response at the site of infection.

During IAV infection, RNA released during cell lysis serve as ligands to activate TLR7^+^ myeloid cells, including macrophages, neutrophils, DCs, and MDSCs. Resident alveolar macrophages are among the first responders to infection [52]. Neutrophils and macrophages are recruited on days 3 and 5 p.i. (Figure 3c). On day 5 p.i., MDSCs are recruited to the site of inflammation (Figure 3c). They then can become activated by the presence of inflammatory mediators like IL-6 and IL-1 [25]. In mice, virus replication is present through day 7, and is cleared by day 10 (Figure 1d), indicating that TLR7 ligands would be present at least through day 10. Our findings suggest that the MDSCs recruited to the lungs starting on day 5 would recognize the presence of RNA through TLR7 and become hindered in their capacity to inhibit the ongoing inflammatory response similar to TLR9 ligand mediated inhibition of MDSC tumor suppression [46] (Figure 9a). It is possible that the sensing of RNA by TLR7 actually leads to further differentiation of MDSCs into a more classical Th1 inducing macrophage. MDSCs are known to have plasticity in their ability to further differentiate into a different type of myeloid derived cell, as has been previously shown with activation from TLR4 activation [26], [53].

It would not be beneficial for the host to have numerous suppressor cells present before the acute infection is cleared. After day 7 p.i., when RNA is no longer in excess, MDSC activity would no longer be inhibited and would then be allowed to suppress the remaining inflammatory response. In a situation where TLR7 is not present, MDSCs would lose their ability to sense the presence of RNA during an ongoing infection. When the MDSCs are recruited on day 5, they would not suspend their suppressive activity in the presence of inflammation [25]. This would lead to accumulation of MDSCs and the inhibition of a Th1 polarized immune response, further aggravating inflammation in an unbalanced manner (Figure 9b).

Together, our findings suggest that TLR7 signaling inhibits the suppressive activity of MDSCs during IAV infection in mice, resulting in a predominant Th1 response. It has yet to be determined what the long term effects of a Th2 polarized response observed in these TLR7^-/-^ mice are on the memory response. It would be interesting to see if this Th2 polarization increases or decreases the recall response to subsequent infections, and what the consequences of this would be on vaccine design. Because of TLR7 ability to promote a Th1 biased response, it is possible that the inclusion of TLR7 ligands may enhance the immunogenicity of influenza vaccines [12], [54].

## Materials and Methods

### Animals

*C57Bl/6* (B6) wild-type mice were purchased from Charles River Laboratories (Wilmington, MA). *TLR7^-/-^* mice on B6 background were a gift from Akiko Iwasaki (Yale University, New Haven, CT) [55] and Regeneron Pharmaceuticals, Inc (Tarrytown, NY) and were bred at Charles River Laboratories. *B6.Cg-Tg(TcraTcrb)425Cbn/J* mice (OT-II) were purchased from Jackson Laboratories (Bar Harbor, ME). Animals were age matched and housed under pathogen-free conditions. Animal research was conducted under the guidance of the CDC's Institutional Animal Care and Use Committee in an Association for Assessment and Accreditation of Laboratory Animal Care International-accredited animal facility.

### Influenza virus

Influenza A/Puerto Rico/8/34 virus (PR8) was propagated by allantoic inoculation of 10-day old embryonated chicken eggs. The viral plaque titer, 50% mouse infectious dose (MID_50_), 50% egg infectious dose (EID_50_), and 50% lethal dose (LD_50_) were determined using methods described previously [56]. One MID_50_ was equivalent to 40 PFU, 10 EID_50_, and 0.01 MLD_50_.

### Infections of mice and harvesting tissues for flow cytometric analysis and viral titer quantification

All mice were infected intranasally (i.n.) with 25 MID_50_ of PR8 under anesthesia in a volume of 50 µL or mock infected with 50 µL of phosphate buffered saline (PBS). Animals were monitored daily for 14 days post-infection (p.i.) for body weight changes and other clinical signs of morbidity. On the indicated days p.i., animals were sacrificed to harvest lungs, spleens, mediastinal lymph nodes (MLN), and sera. To characterize cellular infiltrates, lungs were digested with collagenase A (Sigma-Aldrich, St. Louis, MO) to release cells. Spleens, MLNs, and digested lungs were passed through 70 nm cell strainers (BD Biosciences, San Jose, CA) to make single cell suspensions, treated with ammonium chloride (Sigma-Aldrich, St. Louis, MO) to lyse red blood cells. Cell numbers were then counted using a hemocytometer. Cells were later stained with fluorochrome-conjugated antibodies for flow cytometric analysis, using an LSR-II (BD Biosciences, San Jose, CA). Percent of total cells for various cell types were calculated using the gating strategy demonstrated in Figure S1. Percent total numbers were multiplied by total cell numbers counted to obtain total cell numbers for each cell type indicated. To determine viral titers and cytokine concentrations, lungs were homogenized in 1 ml of cold PBS. Clarified homogenates were titrated in 10–11 day old eggs to determine the EID_50_ viral titers in the lungs as previously described [57].

### Hemagglutination Inhibition (HI) Assay

Serum samples were treated with receptor-destroying enzyme (Denka Seiken Co., Tokyo) overnight at 37°C, followed by heat inactivation (56°C for 30 min). Serially diluted sera in V-bottom 96-well plates were tested in duplicate for their ability to inhibit the agglutination of 0.5% turkey red blood cells by 4 HAU PR8 in a standard hemagglutination inhibition (HI) assay as described previously [58].

### Histopathology

Mice were either infected with 25 mID_50_ PR8 or mock infected with PBS. Mouse tissues were harvested either 3 or 7 days p.i. For histological analysis, lung tissues from euthanized mice were fixed in 10% neutral buffered formalin **(**Fisher Scientific, Pittsburg, PA) for two days and embedded in paraffin. Three-micrometer sections from formalin-fixed, paraffin-embedded specimens were stained with hematoxylin and eosin (H&E) for histopathological evaluation. Inflammation scores were determined by a pathologist.

### Intracellular cytokine staining (ICCS)

Lungs were processed as described above. Cell suspensions were prepared in RPMI media (Invitrogen, Carlsbad, CA) supplemented with 10% fetal bovine serum (HyClone, Thermo Scientific, Hudson, NH), 100 ng/mL Penicillin/streptomycin (Invitrogen, Carlsbad, CA), 50 mM β Mercaptoethanol (Invitrogen, Carlsbad, CA). Cells from lungs were cultured with PR8 at a multiplicity of infection of 1 overnight and treated with Brefeldin A (BD Biosciences, San Jose, CA) for 6 hours. Cells were then stained for surface markers, placed in permeabilization/fixation buffer (BD Biosciences) and stained for fluorochrome conjugated antibodies against mouse cytokines to assess the number of cells expressing cytokines.

### Cytokine/Chemokine analysis by Bio-Plex assay

Following the manufacturer’s protocol, a panel of 23 inflammatory cytokines and chemokines was measured using a Bio-Plex suspension array system (Bio-Rad, Hercules, CA). Briefly, clarified lung homogenates collected from PBS control and infected groups were diluted 1∶2 in PBS. Samples and assay standards were added to a 96-well filter plate, followed by anti-cytokine antibody-coupled beads, biotinylated bead detection antibodies and, finally, phycoerythrin (PE)-conjugated streptavidin. Plates were read using a Bio-Plex suspension array system and data were analyzed using Bio-Plex Manager 4.0 software (Bio-Rad, Hercules, CA).

### *In vitro* functional analysis of MDSCs

B6 and TLR7^-/-^ mice were infected with PR8, and lungs were harvested on day 7 p.i. Lungs were processed for a single cell suspension as described above, and stained with fluorochrome-conjugated antibodies. Gr1^+^CD11b^+^ cells were collected using FACS Aria (BD Biosciences, San Jose, CA). Sorted cells were plated at 1×10^5^cells/well in a 96 well flat bottom plate. Splenocytes from B6 mice were harvested as described above and incubated with 10 nM of OT-II peptide (OVA 329 - 337) (AnaSpec Inc., San Jose, CA) for 1 hour at 37°C, washed three times with RPMI-10 +10% FBS, and plated with the MDSCs at 1×10^5^ cells/well. Untouched CD4+ T cells from the spleens of OT-II mice were purified with MACS Microbeads (Miltenyi Biotec Inc, Auburn, CA) using negative selection. Transgenic CD4+ T cells were plated with the other two cell types at 1×10^6^ cells/well. Plates were incubated for 24 hours at 37°C in a CO_2_ incubator. 18 hours after plating, Brefeldin A was added and cultures were incubated for the remaining 6 hours. Cells were then stained with blue-fluorescent reactive live/dead dye (Invitrogen, Eugene, OR), surface markers, and intracellular cytokines as described above and analyzed using BD LSR-II flow cytometer.

### Antibody isotype determination by ELISA

ELISA plates were coated with 100HAU/well of PR8 overnight at 4°C. Plates were washed 3X with PBS-Tween (PBST) and then blocked with 5% bovine serum albumin (BSA) for 2 hours at room temperature. Plates were washed 3X with PBST. Mouse sera were diluted 1∶10, added to wells, and incubated overnight at 4°C. Plates were washed 3X with PBST, and horseradish peroxidase (HRP) conjugated isotype specific anti-mouse Ig (Southern Biotech, Birmingham, AL) was added at 1∶5000 dilution to each well for 2 hours at room temperature. Plates were washed 3X with PBST and TMB substrate (Southern Biotech, Birmingham, AL) was added to each well, followed by stop solution. Absorbance was read at 450 nm. A standard curve of purified mouse IgM, IgG, IgG1, or IgG2a was used as a measurement of antibody concentration.

### Antibodies for flow cytometric analysis

Fluorochrome-conjugated anti-mouse antibodies against CD3(Pacific Blue, APC), DX5 (FITC), NK1.1(PerCP Cy5.5), CD19 (PE, APC-Cy7, PerCP Cy5.5), B220 (PE-Cy7, Pacific Blue), CD11c (FITC), Gr1 (APC-Cy7), CD11b (Alexa Fluor 700, PerCP Cy 5.5), IFNγ (Alexa Fluor 700), IL-17 (FITC), CD4 (PE, Alexa Fluor 700), CD8 (FITC, PE-Cy7), CD95 (PE-Cy7), and GL7 (FITC) were purchased from BD Biosciences, San Jose, CA. Flurochrome conjugated antibodies against murine F4/80 (APC), IL-10 (APC), TNFα (eFluor 450), and IL-4 (PE-Cy7) were obtained from eBiosciences, San Diego, CA. The PR8 nucleoprotein(NP)-tetramer (H-2D^b^ ASNENMETM) (PE) was purchased from ProImmune, Sarasota, FL. Samples were run on a BD LSRII and data were analyzed using FlowJo 7.5.5 (Treestar, Ashland, OR).

### Statistical analysis

Statistical significance was determined by a two tailed student’s t-test unless otherwise mentioned. Asterisks indicate the levels of statistical significance relative to B6 control: * =  p<0.05, ** = p<0.01,*** = p<0.001. Error bars represent the standard error of the mean (SEM).

## Supporting Information

Figure S1 **Gating strategy for innate infiltrates.** Total cells were gated on SSC vs. FSC. SSC+FSC+ cells were sub-gated into CD3 vs. NK1.1/DX5. CD3+ cells were considered T-cells (1) and NK1.1/DX5 cells were considered NK cells (2). CD3- cells were then sub-gated inot CD19 vs. B220. CD19+B220+ cells were considered B-cells (3). CD19- cells were sub-gated into F4/80 vs. CD11c. F4/80-CD11c+ cells were considered DCs (4). Cells that were not considered DCs were sub-gated into Gr1 vs. CD11b. These are the plots represented in Figure 3a. Gr1+ cells were further sub-gated into SSC vs. F4/80. SSC+F4/80^low^ cells were considered Neutrophils (5) while SSC^low^F4/80+ cells were considered to be MDSCs (6). These are the plots represented in Figure 3b. Gr1- cells were then sub-gated into F4/80 vs. CD11b. F4/80+CD11b+ cells were considered macrophages (7).
(TIF)

Figure S2 **Recruitment of lymphocytes and macrophages to lungs was not significantly affected in TLR7^-/-^ mice**. On indicated days p.i., lungs (n≥5 mice) were harvested, infiltrating cells were stained with appropriate fluorochrome conjugated antibodies. Total cell numbers from two independent experiments were calculated for macrophages (CD11b^+^, Gr1-, F4/80^+^), B-cells (CD19^+^, B220^+^), T-cells (CD3^+^), and NK cells (CD3^-^DX5^+^/NK1.1^+^). (B) A separate experiment tested the recruitment of lung infiltrates on day 10 post infection. Again, lungs (n≥4 mice) were harvested, stained for cell specific markers, and total cell numbers were calculated as described above.
(TIF

Figure S3 **No changes in cytokine expression were observed in splenocytes on day 10 post-infection**. Splenocytes were harvested and ICCS performed as described in materials and methods. One representative histogram of three different mice showing the expression of IL-4, IL-17, IL-10 or IFNγ by (A) CD4+ cells or (B) IFNγ by CD8+ cells is shown.
(TIF)
